# First evidence of the feasibility of gaze-contingent attention training for school children with autism

**DOI:** 10.1177/1362361315617880

**Published:** 2016-02-09

**Authors:** Georgina Powell, Sam V Wass, Jonathan T Erichsen, Susan R Leekam

**Affiliations:** 1 Cardiff University, UK; 2 Medical Research Council (MRC) Cognition and Brain Sciences Unit, UK

**Keywords:** attention, autism, cognitive training, eye movements

## Abstract

A number of authors have suggested that attention control may be a suitable target for cognitive training in children with autism spectrum disorder. This study provided the first evidence of the feasibility of such training using a battery of tasks intended to target visual attentional control in children with autism spectrum disorder within school-based settings. Twenty-seven children were recruited and randomly assigned to either training or an active control group. Of these, 19 completed the initial assessment, and 17 (9 trained and 8 control) completed all subsequent training sessions. Training of 120 min was administered per participant, spread over six sessions (on average). Compliance with the training tasks was generally high, and evidence of within-task training improvements was found. A number of untrained tasks to assess transfer of training effects were administered pre- and post-training. Changes in the trained group were assessed relative to an active control group. Following training, significant and selective changes in visual sustained attention were observed. Trend training effects were also noted on disengaging visual attention, but no convincing evidence of transfer was found to non-trained assessments of saccadic reaction time and anticipatory looking. Directions for future development and refinement of these new training techniques are discussed.

## Introduction

Autism spectrum disorders (ASDs) are neurodevelopmental disorders characterised by impairments in social interaction and communication, and the presence of restrictive, repetitive patterns of behaviour (APA, 2013). Although they are not a diagnostic feature, aspects of atypical attention have frequently been reported to be part of the cognitive profile of ASD (see Ames and Fletcher-Watson, 2010; Keehn et al., 2013; Orekhova and Stroganova, 2014, for review). Furthermore, difficulties have been reported in aspects of volitional attention control not only in social but also in non-social situations (e.g. Benson et al., 2012; Kourkoulou et al., 2013; Leekam et al., 2000).

Importantly, recent research into the emergence of the ASD phenotype suggests that differences in aspects of attention appear very early in ASD symptomatology. Research suggests that difficulties that might be specific to social orienting are *not* generally detectable at very early stages of development (Jones et al., 2014). Instead, infants who later develop ASD are similar to typically developing infants in their orienting to faces (Elsabbagh et al., 2013b; Young et al., 2009) and to social movement (e.g. eyes, mouth or hands movement; Elsabbagh et al., 2013a). However, impairments in domain-general aspects of development, including in aspects of non-social attention, *have* been noted very early in infants who later receive a diagnosis of ASD. For example, Elison et al. (2013) found that infants who later develop ASD are slower to disengage attention at 7-month-old infants, and Wass et al. (2015) found differences in micro-temporal eye movement patterns in 6-month-old infants who later developed ASD (see also Jones et al., 2014).

The ability to exercise control over attention is thought to be a ‘hub’ cognitive faculty – that is, a faculty required for the acquisition of skills in a range of other areas (Karmiloff-Smith, 1992; Posner and Rothbart, 2007; Wass, 2014; Wass et al., 2012). The ability to regulate and direct attention releases the child from the limitation of passively responding to environmental events, which means that they are able to actively guide their attention towards the information-rich areas key for learning (Ruff and Rothbart, 1996; Scerif, 2010). Individual differences in aspects of attentional control have been shown to correlate with early language development (Kannass and Oakes, 2008; Rose et al., 2009) and early learning in academic settings (Razza et al., 2010; Rose et al., 2011; Steele et al., 2012; Welsh et al., 2010). It is thought that early disruptions in aspects of attention control can lead to cascade-like patterns of subsequently impaired learning across a range of domains (Johnson, 2012; Karmiloff-Smith, 2009). Thus, targeting attention training at a young age could facilitate improvements in other areas of development.

A variety of treatment techniques exist for ASDs. Of the non-pharmacological interventions available, currently the largest evidence base exists for early, intensive behavioural interventions (Reichow, 2012). Significant intervention effects have been demonstrated both on core ASD symptomology and on more general cognitive performance (Wallace and Rogers, 2010). However, the staffing costs of these kinds of intervention can be formidable: typically home-based, early interventions commonly last 2+ years and involve clinician care for upwards of 40 h per week.

Relatively, little research, however, has examined the effect of applying targeted cognitive training to individuals with ASD (Wass and Porayska-Pomsta, 2013). Cognitive training regimes target particular, pre-defined cognitive domains. Evidence from cognitive training studies has shown that cognitive training can lead to changes in a variety of non-trained aspects of behaviour, such as learning in academic and other social settings. Moreover, training effects are generally found to be stronger, when training is applied at early stages of development (Wass, 2014; Wass et al., 2012). This study examined the feasibility of targeted cognitive training aimed at improving visual attention control, delivered via a gaze-contingent interface. In gaze-contingent interfaces, participants view a screen that has an eyetracker attached, and different events take place contingent on where on-screen the participant is looking. There is no need to interface via a keyboard or mouse, and no verbal instructions are involved (Wass and Porayska-Pomsta, 2013).

The training interfaces used in this study are based on those developed for typical infants and toddlers by Wass et al. (2011). In this previous study, a battery of gaze-contingent training tasks targeting the cognitive domains of interference resolution, inhibition, task-switching and working memory was administered to typically developing 11-month-old infants across four visits. Training effects were assessed relative to a control group. Immediately after training, improvements in attentional disengagement, saccadic reaction time (RT), cognitive control and visual sustained attention were observed (Wass et al., 2011), as well as marginally non-significant changes in looking behaviour during free play. No changes were found in working memory. Ballieux and colleagues investigated whether these training effects could be replicated with typically developing infants (12-month olds) from low socio-economic status backgrounds (Ballieux et al., in press). The training was administered in community settings. Significant transfer of training improvements was again observed on most but not all tasks.

### The present study

In this pilot study, we wished to evaluate the feasibility of applying gaze-contingent attentional control training paradigms, which previously had been used in both laboratory and community settings with infants, to children with ASD. To do this, we addressed three questions: (1) Are school-based training environments suitable for hosting this kind of repeat-visit training study? (2) Can sufficiently good quality data be collected in these settings, and with these populations? (3) Are training tasks sufficiently difficult and engaging to maintain interest over multiple sessions in children?

We recruited children with ASD aged 3–9 years – including severely impaired as well as less severely impaired individuals. We used similar measures to those included in the original study (Wass et al., 2011). A battery of four gaze-contingent training tasks targeting interference resolution, inhibition, visual search, online goal maintenance and task-switching was administered across a number of visits to the school. Before and after training, a battery of assessment tasks was administered to measure visual sustained attention, saccadic RT, attentional disengagement latencies and anticipatory saccades. The effect of training was assessed relative to a control group, also with ASD, who attended an equal number of sessions, but, instead of training, watched age-appropriate videos and animations while their gaze was monitored by the eyetracker. We expected that, consistent with previous research, training would lead to improvements in a variety of untrained aspects of attentional control. Specifically, we predicted (based on Wass et al., 2011) that the trained group would show (1) a selective increase in looking time in the sustained attention task, (2) reduced attentional disengagement latencies, (3) faster saccadic RTs and (4) faster anticipatory saccades.

## Methods

### Participants

Participants were recruited from three schools in South Wales (one mainstream, two special needs). No exclusion criteria were specified because we wanted the study to be as inclusive as possible. In all, 27 children aged 3 years 7 months to 9 years 11 months were initially recruited to the study. Of these, eight failed to complete the pre-testing battery at either the first or second attempt, and so were excluded from subsequent participation in the study. A further two children (one trained and one control) were subsequently excluded as they failed to complete more than 60 min of training or control sessions. In total, 17 children (9 trained and 8 control) completed the study. Of these, all children had a community multidisciplinary team assessment leading to a best estimate clinical diagnosis of an ASD (including autism and Asperger syndrome) according to *Diagnostic and Statistical Manual of Mental Disorders*, 4th ed. (DSM-IV) and International Classification of Diseases – 10th Revision (ICD-10) criteria.

### Study design

Prior to their first visit, children were randomly allocated into either trained or control groups. Of note, in this small-scale pilot study, the same researcher conducted all testing and pre–post sessions and therefore could not be blinded to group allocation. Table 1 shows a comparison of how the two groups compared on mental age (MA) and calendar age (CA). None of these measures showed significant differences between groups at pre-test. Training and control groups were also compared on assessments of verbal, non-verbal and spatial ability (British Ability Scales (BAS)). Again, no significant differences were found between the groups.

**Table 1.** Participant characteristics.

	Trained	Control
N	9	8
Gender	1 female	1 female
CA	6 years 5 months (17 m)	7 years 2 months (18 m)
MA (BAS and Mullen reported together)	4 years 6 months (21 m)	4 years 9 months (33 m)
BAS – general cognitive ability (composite IQ score)	62.26 (19)	73.86 (36)
BAS – verbal	68.17 (11.3)	86 (27.6)
BAS – non-verbal	71.71 (7.7)	78.43 (18.5)
BAS – spatial	83 (22.3)	89.71 (25.6)

Standard deviations are in parentheses. BAS: British Ability Scales; CA: calendar age; MA: mental age.

At first visit, all children conducted the pre-test battery. This battery, which is described in more detail below, lasted for approximately 20 min. At subsequent visits, children participated in either training or control sessions. Each training and control session lasted for as long as the child remained engaged with the materials presented, following a set procedure that was consistent for all children. Training or control sessions were continued, approximately twice per week, until the child had completed 120 min. After completion of the training or control sessions, the post-test battery was presented at the final visit. The post-test battery was identical to the pre-test battery.

### Materials and procedures

Testing equipment consisted of (1) a Tobii X2-60 portable eyetracker; (2) a 23″ external monitor; (3) a laptop running Windows 7. The X2-60 eyetracker has a temporal resolution of 60 Hz, and a manufacturer-reported spatial resolution of 1°–2° of visual angle (http://www.tobii.com/Global/Analysis/Downloads/User_Manuals_and_Guides/Tobii_X2-60_EyeTrackerUserManual_WEB.pdf). The monitor, with the eyetracker below it, was positioned directly facing the child. The experimenter sat, with the laptop, at approximately 50 cm distance from the child’s coronal plane, subtending 50° of visual angle. All testing and training materials were administered via MATLAB and Psychtoolbox. Calibration was carried out via a standard 5-point calibration procedure, based on an adaptation of the version supplied with the MATLAB SDK. During testing, online tracking accuracy was monitored by an experimenter via a live feedback screen. If tracking was lost or poor, recalibration was occasionally reattempted during a session – although this was performed only relatively infrequently.

All pre–post, training and control sessions were conducted in quiet rooms that were made available within the schools. The researcher visited each school twice a week during the school’s participation in the study.

The BAS (Elliot et al., 1996) was administered to 15 of the 17 participants, in order to assess general cognitive abilities. Of these, 12 participants completed the Early Years scales and three participants completed the School Age scales. Two participants were not able to complete the BAS and so were administered the Mullen Scales of Early Leaning (MSEL), which assess ability across five domains – Gross Motor, Visual Reception, Fine Motor, Expressive Language and Receptive Language. Age equivalent scores on the MSEL were calculated for both children.

### Pre–post tests

**Figure 1.**
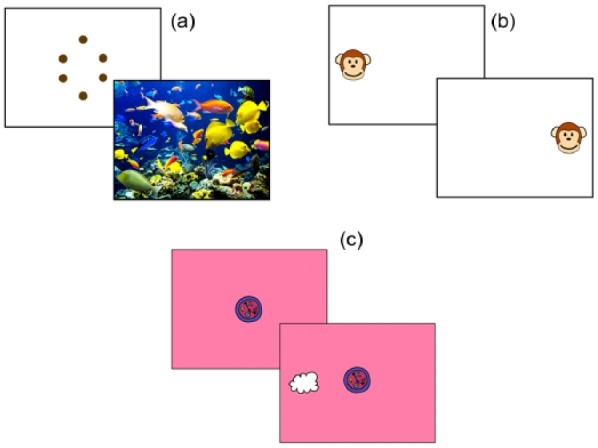
Schematics showing the pre–post tests that were administered: (a) Examples of the ‘boring’ (top) and ‘interesting’ (bottom) stimuli used in the visual sustained attention task. (b) Illustration of the screen layout for the anticipation task (two-location condition). In the four-location condition, objects were presented in the four corners of the screen. (c) Illustration of screen layout for the overlap condition gap-overlap task. In the baseline condition, the central target disappeared as the lateral target was presented. (Colour version available online. DOI: 10.1177/1362361315617880.)

In order to assess transfer of training effects, the following pre–post tasks were presented interleaved (Figure 1). In order to maintain engagement during testing, four short clips from TV programmes were also presented, between experimental blocks.

#### Visual sustained attention

Four different still images were presented, in separate blocks at different stages of the testing protocol. Two of these images were ‘interesting’ (i.e. attractive, detailed images of flowers and fish), and the other two were ‘boring’ (i.e. low-detail, monochrome figures of a diamond and a cross). Based on previous research, we predicted that a selective change in behaviour would be observed, consisting of an increase in looking time to the ‘interesting’ but not to the ‘boring’ images (Wass et al., 2011).

Trials commenced once the participant had fixated a central target (CT). Trials ended when the participant either looked away from the screen for 1 s, as judged by an experimenter (see below), or following 45 s after the trial started. After the end of each trial, a fixation target and brief auditory stimulus (<1 s) were then presented. If the participant looked at the target, the next trial started immediately; if not, a sequence of different fixation targets and auditory attention getters was repeated. Eight trials were presented per stimulus. During the trial, the experimenter viewed a feedback screen, showing whether gaze was detected by the eyetracker. When this feedback screen showed that no gaze was detected, the experimenter visually confirmed that the child was not looking at the screen and then pressed a key to trigger the end of the trial. This protocol was used because occasionally the eyetracker lost contact with the child’s eyes, even while they were looking at the screen. The dependent variable (DV) was first look duration (in seconds), the duration of each child’s first look to the screen following the presentation of a new stimulus.

#### Anticipatory saccades

Four blocks (two blocks of two conditions) were presented at different stages of the testing protocol; each block lasted 12 trials. The two conditions were two targets and four targets. In the two-target condition, a target cycled between two locations on the screen (ABABABABABAB). In the four-target condition, the target cycled between four locations (ABCDABCDABCD). Each trial consisted of a 1000 ms blank inter-stimulus interval (ISI), followed by the appearance of a target. As soon as the child looked at the target, a reward sequence was triggered. If no look was registered within 4000 ms, the trial ended with no reward sequence. RT was coded as the latency between the appearance of the target and the first saccade to the target. If an anticipatory saccade had taken place during the 1000 ms ISI, this was recorded as a negative RT. Participants were excluded if fewer than eight usable trials were registered per condition.

#### Attentional disengagement latencies/saccadic RT

After fixating a CT (a cartoon flower, 4.5°), a lateral target (LT, a cartoon cloud, 3°) was presented to the left or right; when the participant fixated the LT, s(he) received a brief audio-visual reward. Two conditions were presented: baseline – CT disappears concurrently with LT appearance; overlap – CT remains on-screen with LT appearance. This task was presented in three blocks of 12 trials. The RT was the time elapsed between LT appearance and the reported position of gaze leaving the central fixation area (a 9° box around the CT). RTs less than 100 and greater than 2000 ms were excluded. If the participant was still fixating the CT at the end of the 2000 ms window, their response was coded as 2000 ms. Participants from whom fewer than six usable trials per condition were obtained were excluded from further analyses. Of note, this threshold is lower than the threshold of 12 usable trials per condition that was used in previous research. Setting a lower threshold was necessitated by the high rates of data drop-out observed on this task (see Supplementary Materials). Disengagement latencies were calculated as the participant’s average RT in the overlap condition subtracted from their average RT in the baseline condition (Elsabbagh et al., 2009).

### Training battery

**Figure 2.**
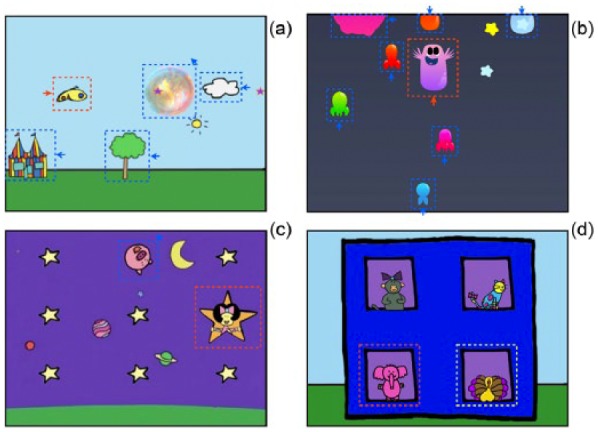
Schematics of the four training tasks administered. Dashed rectangles and arrows, which were not visible in the original tasks, indicate objects that were moving on-screen: (a) Task 1 (Butterfly). The butterfly (indicated in red) scrolled from left to right as long as the child looked directly at it, with static and moving (indicated in blue) distractors presented in the child’s peripheral visual field. If the child looked to any of the distractors, they disappeared and the scrolling stopped. (b) Task 2 (FlyMe). The purple character (indicated red) scrolled upwards as long as the child looked directly at it. Static and moving (indicated blue) distractors appeared from the top and bottom of the screen. If the child looked to any of the distractors, the purple object sank towards the bottom of the screen and the distractors disappeared. (c) Task 3 (Stars). A target (indicated red) was presented on-screen along with a number of static and moving (indicated blue) distractors. If the child looked to the target within a time window, (s)he received a reward. Both target and distractors changed between trials. (d) Task 4 (Suspects). A target (indicated red) was presented along with a range of distractors. If the child looked to the target within a time window, they received a reward. Once per block of 12 trials, the target changed. Targets from the previous block (indicated yellow) were presented concurrently with the current target, as distractors. (Colour version available online. DOI: 10.1177/1362361315617880.)

The training battery consisted of four different training tasks, targeting a combination of interference resolution, inhibition, visual search, online goal maintenance and task-switching (Figure 2). These were presented in rotation, in an order that was counterbalanced between visits and between participants. All four tasks were presented at each training session, until the participant refused to engage further with that task. A fifth training task was also initially included, but excluded within the first 2 months of the study as it proved insufficiently engaging to children. This task is Task 3 (Windows), as described in Wass et al. (2011).

The following four tasks were used in this study:

*Task 1 (Butterfly)*. A target (a butterfly) (subtending 6°) was presented on the screen. When the child fixated the target, the butterfly ‘flew’ across the screen, and distractors (a house, a tree, clouds, 5–15°) scrolled in the opposite direction. When the child looked to one of the distractors, they disappeared and only the target, now static, remained on-screen. Once the target was re-fixated, it re-commenced moving and the distractors re-appeared and continued scrolling. The salience of the distractors changed adaptively, including faster, larger and more densely packed objects. This task rewards a child for maintaining their fixation on one target, and suppressing the pre-potent response to look towards moving distractors in the periphery.
*Task 2 (FlyMe)*. A target (a purple character) (subtending 6°) was presented on the screen. When the child fixated the target, the character ‘flew’ upwards, and a number of distractors, such as clouds, appeared from the top of the screen. When the child looked to any of the distractors, the character began rapidly to sink towards the bottom of the screen and the distractors disappeared from the top of the screen. As the character ‘flew’ higher, the background became darker and the distractors more salient, including rockets, planets and stars. At higher difficulty levels, a further one or two flying characters were presented concurrently (one purple and one red); each ‘flew’ upwards only when the child looked directly at it. Therefore, the child had to shift their attention from looking towards one target to the other (looks to the distractors were never rewarded). At lower difficulty levels, this task therefore encourages a child to maintain their fixation on one target while suppressing the pre-potent response to look towards moving distractors in the periphery. At higher difficulty levels, it rewards shifting fixation between two or three targets while ignoring peripheral distractors.
*Task 3 (Stars)*. One of five possible targets (a cartoon character placed within brightly coloured stars) (6°) was presented on-screen together with eight distractors (smaller stars, planets, clouds) (4–8°) against a detailed still image as background. If the participant fixated the target within 3000 ms, they received an animation as a reward. The target changed from trial to trial. The salience of the distractors changed adaptively; at lower difficulty levels, the eight distractors were smaller, static, identical to each other and dissimilar to the targets; at higher difficulty levels, they were more varied, moving, more brightly coloured and more similar to the targets. This task requires flexible search for changing targets, while ignoring distractors.
*Task 4 (Suspects)*. One of two possible targets (either an elephant or a chicken) (subtending 4.5–8°) was presented with one or more distractor items of the same size. When the child looked at the target within a time limit, (s)he received an animation as a reward. The same target was then re-presented with other distractor(s). The number of distractors varied adaptively with performance; at higher performance levels, more distractors were presented. Between blocks of 12 trials, the target changed – where previously the child had received a reward for looking to the elephant, they now were rewarded for looking to the chicken. At higher difficulty levels, the target from the previous block was presented concurrently with the target from the current block (a conflict trial); at lower difficulty levels, only novel distractors were presented (non-conflict). This task requires flexible search for changing targets, while ignoring distractors.

### Control group stimuli

Control sessions were conducted in the same room, with the same experimenter and using the same eyetracker as the training sessions, and had the same duration and spacing (yoked to a trained participant). Instead of training, control participants viewed a selection of age-appropriate TV clips and still images. Many of these were identical to those used in Wass et al. (2011), but some new videos were added to reflect the older age range of participants.

## Results

### Feasibility

Of the children who completed the study, the number of sessions needed to complete the 120 min required was 6.3 (standard deviation (SD) = 0.87, range = 5–8) sessions for the trained group and 6 (SD = 1.07, range = 5–8) for the control group. The average time between the first and last testing sessions was 41.4 (SD = 11.9, range = 24–59) days for children in the trained group, and 36.5 (SD = 13.8, range = 21–60) days for children in the control group.

In order to evaluate the quality of raw eyetracking data obtained on this trial, which used eyetrackers within school settings, a comparison was conducted between data obtained in this study and data from a previous study of typical 12-month-old infants conducted in laboratory settings, using a Tobii 1750 eyetracker (Wass et al., 2011). The experimental paradigms used in the datasets were identical – namely, the gap-overlap experiment. Data quality evaluations were calculated using techniques described in detail in Wass et al. (2014).

**Figure 3.**
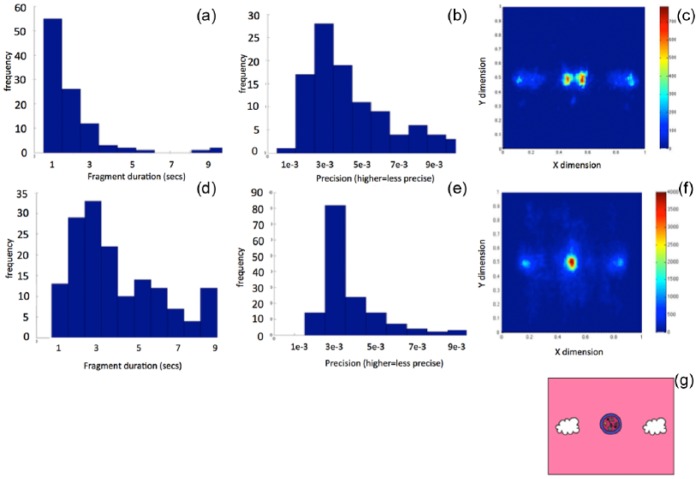
Data quality comparison based on data from the gap-overlap study: (a–c) data from this study, (d–f) data from a comparison study that used identical procedures, in laboratory settings, with typical infants (Wass et al., 2011). (a) and (d) Histograms showing the duration of usable fragment durations that were present in our data (calculated on a block-by-block basis). Markedly, longer usable fragment durations were obtained in the comparison study. (b) and (e) Histograms showing the precision of our data (calculated on block-by-block basis). Markedly, more precise data were obtained in the comparison study. (c) and (f) Gaze maps of usable gaze data obtained during the trial. (g) A schematic of how images were distributed on the screen during the trials. (Colour version available online. DOI: 10.1177/1362361315617880.)

Two measures of data quality have been presented. First (Figure 3(a) and (d)), the robustness of tracking was quantified by calculating the duration (in seconds) of usable fragment durations obtained during recording. A low number indicates that data tended to ‘flicker’ on and off during recording. This is a common problem during remote eyetracking that has been described in detail elsewhere (Leppänen et al., 2014; Wass et al., 2014). Markedly, shorter usable fragment durations were obtained in this study relative to the comparison study. The average (standard error of the mean (SEM)) was 1.4 (0.17) s in this study, and 3.7 (0.26) s in the comparison study. Second (Figure 3(b) and (e)), the precision of tracking was quantified by quantifying the degree to which reporting of position of gaze is consistent between samples. A higher value indicates that data obtained were less precise. Less precise data were obtained in this study relative to the previous study. The average (SEM) was 3.8e–03 (0.4e−03) in this study, and 3.2e–03 (0.1e−03) in the comparison study (internal units). Third (Figure 3(c) and (f)), all available data obtained during recording of the two experiments were visualised using a standard heat map technique. Figure 3(g) shows, for comparison, where on-screen information was located in this task. Data for the two studies appear approximately equivalent. In conclusion, therefore, substantially less precise and more flickery data were obtained in this study relative to the comparison study. However, the accuracy of reporting of position of gaze between the two studies appears approximately equivalent.

For each measure, bivariate correlations between pre- and post-test performance were also calculated within this study, using Pearson’s r. This gives an estimate of the stability of that measure as an index of individual differences, ignoring training-associated changes. The results indicated reasonably high consistency: first look to ‘interesting’ (r = 0.52); first look to ‘boring’ (r = 0.43); disengagement latency (r = 0.44); average saccadic RT (r = 0.58); anticipatory saccades (2-object) (r = 0.64); anticipatory saccades (4-object) (r = 0.48).

### Training responsiveness

The mean (SD) playing time per task was as follows: Task 1 (Butterfly) 203 (113) s; Task 2 (FlyMe) 158 (134) s; Task 3 (Stars) 263 (93) s; Task 4 (Suspects) 289 (83) s. The total duration of each training session remained approximately constant across the study, at 15–20 min per session.

**Figure 4.**
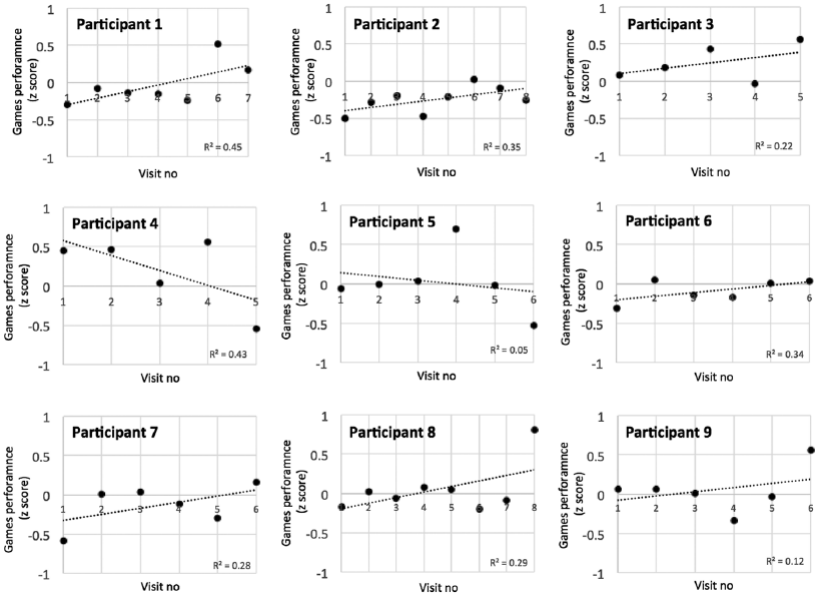
Scatterplots showing, participant by participant, how performance on the training tasks changed across the course of the training sessions. Best-fit linear regression lines have been superimposed onto the scatterplots.

Performance within the training tasks was calculated on a per-participant, per-visit basis and re-expressed as z-scores in order to combine results across training tasks. Figure 4 shows, participant by participant, how performance changed across the training visits. A variable number of training sessions were conducted because participants took a variable amount of time to complete the required 120 min of training. Linear regression lines were plotted on this data. Seven of nine participants showed positive gradients, indicating that they improved over the course of the training sessions. This finding was significant (t(8) = 1.89, p(one-tailed) = 0.047) and consistent with predictions based on previous studies (Wass et al., 2011).

### Transfer of training effects – change on pre–post measures

**Figure 5.**
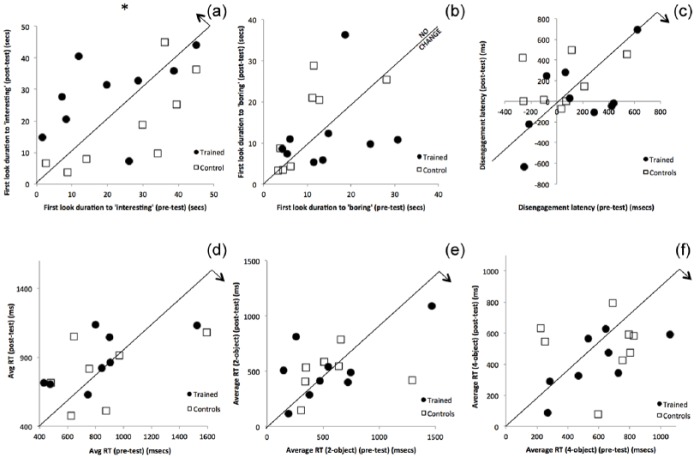
Scatterplots showing change in performance in our participants. Individual dots represent individual children. In each case, pre-test performance has been drawn on the x-axis and post-test performance on the y-axis. A 1:1 equivalence line (indicating that performance at pre-test was identical to post-test) has been drawn on each figure. The direction of predicted change following training, based on previous research, has been shown using an arrow in the top-right corner of each graph. (a) Visual sustained attention – first look duration to ‘interesting’ targets. (b) Visual sustained attention – first look duration to ‘boring’ targets. (c) Disengagement latencies. (d) Average saccadic reaction time (gap-overlap task). (e) Anticipations (two-object task). (f) Anticipations (four-object task).

Table 2 shows mean test performance for the pre–post assessments we administered. All effects observed on mean scores were found to be directionally consistent with predictions based on Wass et al. (2011), and therefore, one-tailed p values were used. Figure 5 shows scatterplots of how performance on our pre–post tasks changed, measure by measure.

**Table 2.** Mean test performance for pre–post assessments.

	Mean (SEM)			
	Trained		Control	
	Pre-test	Post-test	Pre-test	Post-test
A. *Sustained attention*: ‘interesting’ static – first look duration (s)	21 (5)	28.3 (4)	26.3 (6)	19.1 (5)
A. *Sustained attention*: ‘boring’ static – first look duration (s)	14.3 (3)	11.9 (3)	10.1 (3)	14.4 (4)
B. *Gap-overlap task*: disengagement latencies (ms)	155 (45)	25 (122)	45 (16)	210 (80)
B. *Gap-overlap task*: Avg RT (ms)	829 (118)	813 (91)	793 (281)	794 (300)
C. *Anticipation*: two-object (Avg RT (ms))	548 (135)	518 (95)	584 (130)	489 (74)
C. *Anticipation*: four-object (Avg RT (ms))	580 (84)	414 (60)	617 (93)	515 (78)

SEM: standard error of the mean; RT: reaction time.

#### Visual sustained attention

Two DVs were extracted from this task: first look duration to ‘interesting’ stimuli and first look duration to ‘boring’ stimuli. At pre-test, these two measures showed a reasonably strong relationship: r(17) = 0.43, p = 0.07. Based on previous work (Wass et al., 2011), we predicted that training would lead to an increase in look duration to ‘interesting’ stimuli, with no change in look duration to ‘boring’ stimuli. To assess this, we conducted an analysis of covariance (ANCOVA) with the factor group (trained vs control), post-test scores as the DV, and pre-test scores as the covariate. An ANCOVA revealed a significant increase in first look duration to ‘interesting’ stimuli following training F(16) = 5.2, p(one-tailed) = 0.019. No significant change was found for first look duration to ‘boring’ stimuli following training F(16) = 1.2, p = 0.30.

#### Gap-overlap

Two DVs were derived from this task: disengagement latencies and average saccadic RT. Marked differences (shorter disengagement latencies) were observed in this study relative to the previous study with infants (Supplementary Figure S1). Of note, however, this may relate to the data quality issues documented in Figure 3, as previous work has suggested that both of these measures are highly sensitive to differences in the quality of raw eyetracking data obtained (Leppänen et al., 2014; Wass et al., 2014). Table 1 shows that training effects observed were in the same direction as predicted, based on previous research (Wass et al., 2011). To assess the significance of these changes, we conducted and ANCOVA with the factor group (trained vs control), post-test scores as the DV, and pre-test scores as the covariate. Disengagement latencies were shorter for the trained relative to the control group, but this difference did not reach significance (F(15) = 3.00, p = 0.075). No significant change in average saccadic RT was observed as a result of training (F(15) = 0.96, p = 0.17).

#### Anticipation

Anticipations (i.e. saccades that were registered during the 1000 ms anticipatory window before the appearance of the stimulus) were recoded as negative values (up to −1000 ms). The average RTs we observed were 534 ms (i.e. after the stimulus appeared) in the two-object condition and 531 ms in the four-object condition, with no single child scoring a mean negative value. Table 1 shows that training effects observed were in the same direction as predicted, based on previous research. To assess the significance of these changes, we conducted and ANCOVA with the factor group (trained vs control), post-test scores as DV, and pre-test scores as the covariate. No significant changes were observed as a result of training, in either the two-object (F(16) = 0.006, p = 0.47) or four-object conditions (F(16) = 0.11, p = 0.37).

## Discussion

This pilot study assessed the feasibility of administering gaze-contingent training to children with ASD within school settings. We found that, of those children who were able to complete the pre-testing assessment (19 out of 27), all but two were able to complete the entire training protocol. Evidence of improvement within the training tasks was also identified. However, we also noted a number of ways in which training tasks were either too easy, too hard or insufficiently engaging for children in this population – particularly given the heterogeneity of our present sample. This is frequently a problem in the provision of ASD interventions, but can be alleviated if enough time is available to develop more individually tailored tasks. Future work with larger sample sizes and more comprehensive assessment of ability levels will help to identify whether training had a larger effect on children with more, or less, severe general cognitive impairments (Soderqvist et al., 2012).

This feasibility study also noted a variety of practical challenges associated with delivering this training. Although conducting training in a school setting has the advantage of providing high-intensity training at relatively low cost, it was necessary to remove children, one by one, from their normal activities in order to partake in the study, which may have been disruptive. Of note, the quality of eyetracking data obtained was also markedly lower in this study relative to previous studies (Figure 3), which impacted both the responsiveness of the training tasks and the accuracy of the pre–post assessments. Future technological development will, we hope, improve the accuracy of eyetracking in community settings, which will increasingly alleviate these problems.

Despite these challenges, however, evidence of some transfer of training improvements to non-trained tasks was also identified – even given the limitations of this study (primarily the small sample size). We found significant transfer of training improvements to a non-trained assessment of visual sustained attention. This was observed to be selective, insofar as it consisted of changes in looking behaviour towards ‘interesting’ targets but not to ‘boring’ targets. This suggests that the effect of training has been not just to increase the amount of time that children look towards the images, but also to increase the attention that they pay to interesting but not to boring targets. Of note, we found strong relationships between this measure and MA at pre-test (r = 0.52).

Although limited, this finding is encouraging insofar as the previous literature on cognitive training in ASD suggests that it is extremely difficult to demonstrate transfer of training effects following training (Wass and Porayska-Pomsta, 2013). Studies that attempt directly to train social attention in ASD using computerised training have consistently found that training improvements within the training paradigm fail to generalise to other contexts (Faja et al., 2008; Golan and Baron-Cohen, 2006; Swettenham, 1996; Swettenham et al., 1998; Tanaka et al., 2012). The task that we used to assess visual sustained attention is cognitively dissimilar to the training tasks, so the demonstration of predicted transfer is encouraging.

However, it is other tasks, such as disengaging attention, that are widely considered to be more important within the cognitive phenotype of ASD (Bedford et al., 2012). On these, we found no significant changes in our RT tasks following training. Trend (p = 0.075) reductions were found for disengagement latencies, but saccadic RTs and anticipatory saccades did not show convincing evidence of training effects. This is in contrast to previous research, where training was found to lead both to changes in visual sustained attention *and* to changes on RT tasks (Ballieux et al., in press; Wass et al., 2011).

There are four possible reasons for this. First, it could be that the gap-overlap and anticipation tasks were both subject to large amounts of data loss in this study, which obscured genuine training effects (Figure 3). Our findings of both a relatively small disengagement effect relative to previous research, and of relatively slow anticipatory RTs, are consistent with predictions of how eyetracker-based RT measures can be affected by high rates of data loss (Leppänen et al., 2014; Wass et al., 2014), and future work should investigate this area in more detail. Second, it could be that the mechanisms subserving performance on the visual sustained attention task are different to those subserving performance on the other tasks. In previous research, in which concomitant changes in visual sustained attention and RT tasks were observed, it could be that two separate mechanisms were being trained – only one of which was improved in this study. Third, and possibly related, children in this study were markedly older than in previous studies, and increased age may be associated with increased fractionation of attention control mechanisms (Johnson, 2010). Fourth, our relatively small sample size may have masked some training effects that were less pronounced. Children with ASD are known to be extremely heterogeneous, and it is possible the training is effective for some but not others. Indeed, looking at the data at a more descriptive individual level, some participants improved following training while others did not on the tasks where no overall group effect of training was observed. It may be important to explore these individual differences further, with the aim of predicting response to training in the future. Future work with larger sample sizes is required to investigate these important areas in more detail.

In addition to investigating whether our present findings can be replicated with a larger sample size, it will also be interesting to assess the wider transfer of training improvements. This study only evaluated transfer of training improvements to a battery of non-trained assessments of attention control. In future, it will be important additionally to assess whether training effects transfer to aspects of social attention in more naturalistic settings – as initial findings with typical participants suggest (Wass et al., 2011). Finally, it will be desirable to repeat this study with a larger and more homogeneous sample, to assess questions of whether training effects observed are stronger for some children (e.g. the more severely impaired) than for others.

Overall, the published literature suggests that it is desirable to try to apply cognitive training to individuals with ASD, in order to target ‘hub’ cognitive domains such as attention control. However, virtually no previous research has attempted this. The present results are limited, given the small sample size. However, they suggest that it may be feasible to train some of these cognitive abilities in children with ASD using gaze-contingent technology. These techniques have potential practical advantages over clinician-mediated interventions – insofar as they can potentially be applied to a wide range of children, providing intensive training interventions at relatively low cost. However, further work is required to replicate the effects observed in this study, to develop training paradigms that are better targeted towards maintaining engagement and motivation in this population (whose interests are often markedly different to those found in typical children), and also to investigate the effects of applying more extensive assessment of transfer training effects. To this end, the new eyetrackers currently coming onto the market, which cost as little as $100 per unit, may be useful insofar as they open the possibility of home testing. If this work is successful, these technologies have the long-term potential to be integrated as a component of more traditional, clinician-mediated interventions.

## Supplementary Material

Supplementary material
