# Supplementary material for: First evidence of the feasibility of gaze-contingent attention training for school children with autism
Source: Autism. 2016 Feb 9;20(8):927–37. doi: 10.1177/1362361315617880 (PMC5070492; doi:10.1177/1362361315617880)
Supplement: Supplementary material [file AUT617880_Supplementary_file.pdf]

<u>**Supplementary Materials**</u>

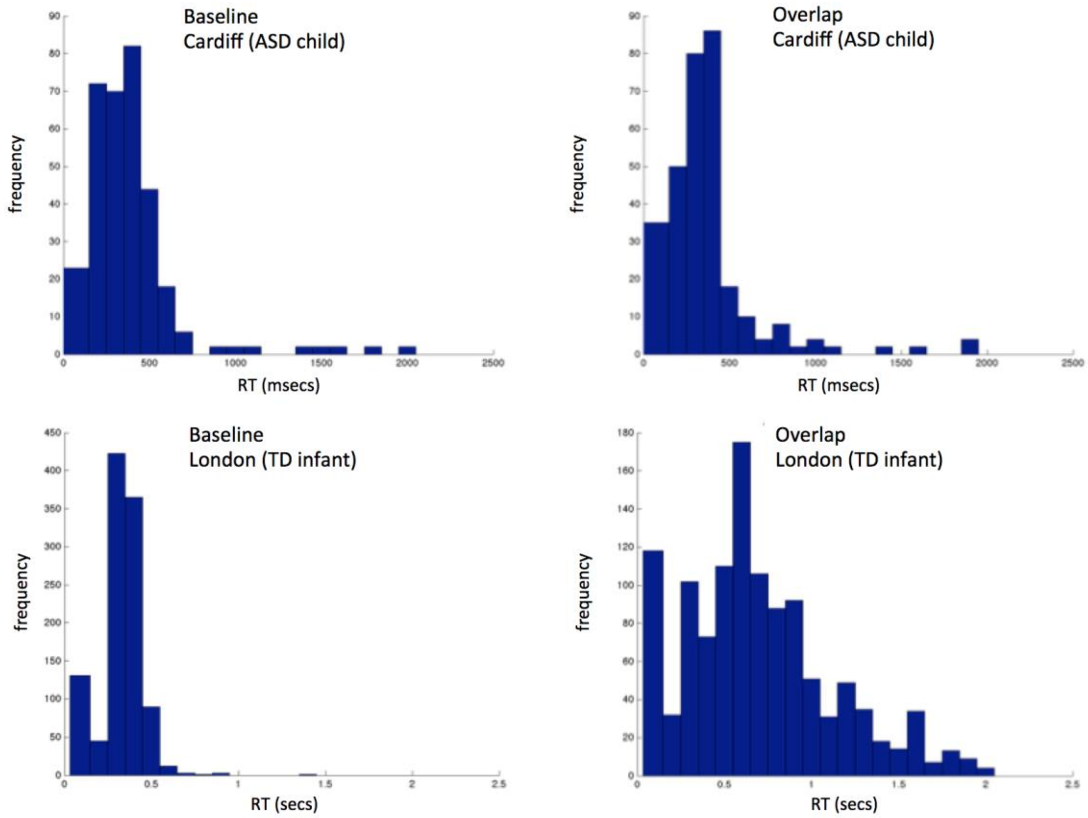

Figure S1: Trial by trial data from the gap-overlap task. Raw data obtained from the two conditions is presented. Disengagement latencies are calculated, participant by participant, as the difference between average RTs obtained in the two conditions (Overlap-Baseline).
